# Body composition, resting energy expenditure and inflammatory markers: impact in users of depot medroxyprogesterone acetate after 12 months follow-up

**DOI:** 10.1590/2359-3997000000202

**Published:** 2016-08-31

**Authors:** Gisele Almeida Batista, Aglécio Luiz de Souza, Daniela Miguel Marin, Marina Sider, Vaneska Carvalho Melhado, Arlete Maria Fernandes, Sarah Monte Alegre

**Affiliations:** 1 Faculdade de Ciências Médicas Universidade Estadual de Campinas Campinas SP Brasil Faculdade de Ciências Médicas, Universidade Estadual de Campinas (FCM-Unicamp), Campinas, SP, Brasil

**Keywords:** Obesity, body composition, resting energy expenditure, depot medroxyprogesterone acetate, leptin

## Abstract

**Objective:**

The aim of this study was to evaluate for 12 months the changes of body weight using Depot Medroxyprogesterone Acetate (DMPA) and if these changes are related to inflammatory markers.

**Subjects and methods:**

Twenty women of childbearing age who chose the DMPA, without previous use of this method, BMI < 30 kg/m^2^, and 17 women using IUD TCu 380A, participated in the study. At the baseline and after one year, changes in weight gain, body composition by the bioimpedance electric method, resting energy expenditure (REE) by the indirect calorimetry method, inflammatory markers and HOMA-IR were assessed.

**Results:**

After 12 months of evaluation, we could observe a significant increase in the DMPA group in weight (3,01 kg) and BMI, while the IUD group’s only significant increase was observed in the BMI. Relative to REE there was an increase of basal metabolic rate (BMR) in both groups after one year. The sub-group DMPA that gained < 3 kg had increased significant weight, BMI and body surface (BS) with respiratory quotient (RQ) reduction, while the sub-group that gained ≥ 3 kg had a significant increase in weight, BMI, BS, fat-free mass, fat mass, BMR, Leptin, HOMA-IR and waist circumference, with RQ significantly reduced.

**Conclusion:**

Our study found significant changes in weight, body composition and metabolic profile of the population studied in the first 12 months of contraceptive use. These changes mainly increased body weight, leptin levels and HOMA-IR which can contribute to the development of some chronic complications, including obesity, insulin resistance and diabetes mellitus.

## INTRODUCTION

Obesity is considered a serious public health problem that affects developed countries as well as developing ones. Globally, there are an estimated 1.5 billion overweight adults, of which approximately 500 million are obese ( 1 ). In Brazil, 50.8% of the population over 18 years old are overweight and 17.5% are obese ( 2 ). This complex and chronic disorder of multifactorial etiology is the result of positive energy balance, and genetic and environmental factors are involved in developing this metabolic disorder ( 3 , 4 ). In women, the use of hormonal contraceptives has been associated with weight gain and changes in body composition.

Depot medroxyprogesterone acetate (DMPA) is an injectable contraceptive method and in its formulation contains 150 mg of DMPA administered every 3 months intramuscularly at a plasma concentration of approximately 1 ng/mL ( 5 ). According to the characteristics of contraceptive efficacy, safety and low cost, and ease of access due to its ready availability from public health systems in several countries, including Brazil, millions of women choose to use this method ( 6 ). Despite having a potential negative effect on bones, the most common reason for discontinuing use of DMPA is increased body weight ( 7 ). Most prospective studies that have assessed the body composition of these progestin users, with at least 30 months of use, show a weight gain associated with an increase in body fat deposits ( 8 , 9 ).

In a study with HIV-infected women was evaluated the association between DMPA use with inflammatory markers ( 10 ). However, no studies in healthy women linking weight gain after using DMPA with inflammatory markers. Therefore, the aim of this study was to evaluate for 12 months the changes of body weight using DMPA and if these changes are related to inflammatory markers.

## SUBJECTS AND METHODS

### Subjects

This was a prospective cohort study with women attending the family planning clinic at the Gynecology and Obstetrics Department, University of Campinas (Unicamp), Brazil. Sample size was calculated from the demand for women seen in the clinic who opted for the contraceptive DMPA method and the percentage of women who have segment to method use for at least 12 months. The women were selected from February 2011 to March 2012.

Women of childbearing age who chose the injectable contraceptive DMPA without previous use of this method, body mass index (BMI) < 30 kg/m^2^, and women using the non-hormonal contraception copper intrauterine device (TCu 380A IUD), were invited to participate in the study. The participants were aged between 18 and 40 years. The groups were paired at baseline, becoming a homogeneous group in relation to age (± 1 year), and BMI (± 1 kg/m^2^).

After signing an informed consent form, they were interviewed to collect data and personal medical and family history. An administration every three months of 150 mg of DMPA was given and follow-up was maintained for one year. The women in the control group, TCu 380A IUD users, underwent the same procedures provided for inclusion into the DMPA group.

Exclusion criteria were: women with diabetes mellitus (DM) type 1 or 2; those with a family history of diabetes; those who had fasting glucose > 100 mg/dL after 8 hours of fasting or after a 120 minute oral glucose tolerance test had 75 g glucose blood glucose > 140 mg/dL; women breast-feeding; those with co-morbidities that contribute to the variation in body weight such as hyper- and hypothyroidism; chronic renal failure and transplant of any organ; use of medications that may be related to weight gain and/or development of insulin resistance such as the chronic use of corticosteroids, antipsychotics, diuretics and statins. Patients with hirsutism and/or hyperandrogenism and polycystic ovary syndrome were also excluded.

**Figure 1:**
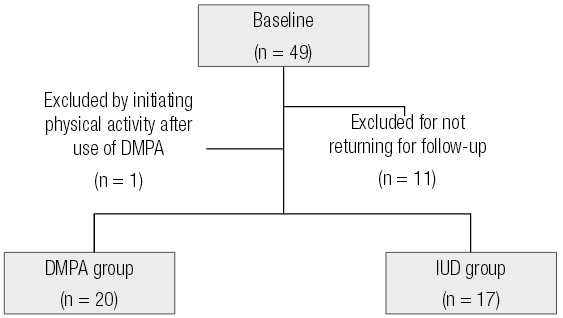
Flow chart of the study.

Initially, 49 women were evaluated. Throughout the monitoring period, 11 volunteers were excluded either for not returning for follow-up or because they became pregnant. One participant was excluded from the group by initiating physical activity after use of DMPA. At the end of the study we evaluated 37 women, 20 from the DMPA group and 17 from the IUD group ( Figure 1 ).

This study was approved by the Ethics Committee of the Faculty of Medical Sciences – Unicamp, and all the volunteers who agreed to participate signed a consent form.

### Anthropometric measurements and body composition

Anthropometric assessment was performed by a single examiner. Weight (kg) and height (m) were measured using a Welmy mechanical scale with 100 g precision and capacity of 200 kg. Height was measured by a stadiometer coupled to the scale with a precision of 0.5 cm. Waist circumference (WC) was measured with an inelastic 2 m tape dimension. The anatomical point used was the midpoint between the last rib and the iliac crest.

Nutritional status was assessed by calculating the BMI, recommended by the World Health Organization 1995 and 1997 ( 11 ).

The percent body fat (% fat), weight, fat mass (FM) and fat-free mass (FFM) were assessed by Bioimpedance Electric (BIA) using the equipment Biodynamics – Bioimpedance Analiser model 310.

### Resting energy expenditure

The resting energy expenditure (REE), basal metabolic rate (BMR) and substrate used to obtain energy were assessed by the indirect calorimetry method. For this, a Vmax Encore 29 device coupled to a canopy gas exchange was used.

The consumption of oxygen (O_2_) and the elimination of carbon dioxide (CO_2_) in liters per minute as well as tidal volume were reported in this procedure. From these primary measures, the values of energy expenditure in kilocalories per day were obtained along with the respiratory quotient (RQ). The mean values obtained in steady state, prefixed as smaller variations than 5% in RQ and 10% in CO_2_ were considered.

### Biochemical assays

Serum adiponectin, interleukin (IL) – 6, visfatin, leptin, tumor necrosis factor-α (TNF-α), insulin and glucose were performed at baseline and after one year of using the method. The blood samples were centrifuged, and serum was immediately stored in small aliquots in a freezer at -80°C. Dosages of adipocytokines and insulin were performed in serum by enzyme immunoassay in duplicate by enzyme-linked immunosorbent assay (ELISA) using commercial kits with high sensitivity and specificity.

### Insulin resistance

Homeostasis model assessment of insulin resistance (HOMA-IR) was calculated according to the formula: fasting insulin (µU/L) x fasting glucose (nmol/L)/22.5. Those considered normal values are < 2.7 ( 12 ).

### Statistical analysis

All results are expressed as mean ±SD. The Mann-Whitney test was used to compare the variables between two groups. The Wilcoxon test was used for comparison of group time fixing. For comparison of numerical measurements between times and groups ANOVA was used for repeated measurements with transformation stations. The significance level for statistical tests was 5% (P < 0.05). The SAS program for Windows version 9.2 was used to perform the analyses.

## RESULTS

After 12 months of evaluation, we could observe a significant increase in the DMPA group in weight (61.95 kg ± 9.69 x 64.96 kg ± 9.40, p = 0.0007), BMI (23.74 kg/m^2^ ± 3.47 x 24.88 kg/m^2^ ± 3.43, p = 0.0007) and body surface (BS) (1.65 ± 0.14 x 1.69 ± 0.13 p = 0.0148), while the IUD group’s only significant increase was observed in the BMI variable (24.26 kg/m^2^ ± 2.67 x 24.56 kg/m^2^ ± 2.84, p = 0.0007) ( Table 1 ).

**Table 1:** Characteristics of the DMPA and DIU groups

Variable	Baseline		¥ P value	12 months		¥ P value
	
	DMPA (n = 20)) Mean ± SD	IUD (n = 17) Mean ± SD		DMPA (n = 20) Mean ± SD	IUD (n = 17) Mean ± SD	
Age (years)	29,7 ± 6,2	28,4 ± 5,8	0,4682	_	_	
Weight (kg)	61,95 ± 9,69	61,24 ± 6,98	0,5449	64,96* ± 9,40	61,76 ± 7,53	0,0007
BMI (kg/m^2^)	23,74 ± 3,47	24,26 ± 2,67	0,911	24,88* ± 3,43	24,56** ± 2,84	0,0007
BS	1,65 ± 0,14	1,62 ± 0,09	0,4127	1,69* ± 0,13	1,63 ± 0,10	0,0148
WC (cm)	75,47 ±7,28	77,61 ± 7,88	0,0178	76,74 ± 6,27	75,38 ± 5,66	0,1834

¥: Mann-Whitney test. Statistical significance: p < 0,05. * Depo group difference between baseline and after 12 months. ** DIU group difference between baseline and after 12 months. BS: body surface; WC: waist circumference; BMI: body mass index.

Relative to REE and RQ, there was an increase of BMR in both groups after one year (DMPA group: 1087.05 kcal/d ± 252.06 x 1319.30 kcal/d ± 133.53, p = 0.0027, IUD group: 1187.59 kcal/d ± 246.23 x 1303.18 kcal/d ± 104.02, p = 0.0027). The RQ was significantly reduced after 12 months of study in groups DMPA (0.91 x 0.77, p < 0.0001) and IUD (0.87 x 0.78, p < 0.0001) ( Table 2 ).

**Table 2:** Resting energy expenditure and respiratory quotient baseline and after 12 months

Variable	Baseline		¥ P value	12 months		¥ P value
	
	DMPA (n = 20) Mean ± SD	IUD (n = 17) Mean ± SD		DMPA (n = 20) Mean ± SD	IUD (n = 17) Mean ± SD	
BMR (kcal/d)	1087,05 ± 252,06	1187,59 ± 246,23	0,3674	1319,30* ± 133,53	1303,18** ± 104,02	0,0027
RQ	0,91 ± 0,11	0,87 ± 0,09	0,8134	0,77* ± 0,03	0,78** ± 0,03	< 0,0001

¥: Mann-Whitney test. Statistical significance: p < 0,05. * Difference Depo group between baseline and after 12 months. ** Difference IUD group between baseline and after 12 months. RQ: respiratory quotient; BMR: basal metabolic rate.

Table 3 shows the subdivision of the DMPA group for weight gain after 12 months follow-up. The sub-group that gained < 3 kg had a significant increase in weight (61.24 kg ± 10.38 x 62.51 kg ± 9.95, p = 0.0156), BMI (23.44 kg/m^2^ ± 4.25 x 23.93 kg/m^2^ ± 4.15, p = 0.0313) and BS (1.64 ± 0.12 x 1.66 ± 0.12 p = 0.0313) with RQ reduction (0.87 ± 0.09 x 0.77 ± 0.03, p = 0.0313), while the sub-group that gained ≥ 3 kg had a significant increase in weight (61.1 kg ± 8.63 x 66.63 kg ± 8.48, p = 0.002), BMI (23.47 kg/m^2^ ± 3.50 x 25.55 kg/m^2^ ± 3.46, p = 0.002), BS (1.64 ± 0.11 x 1.71 ± 0.11, p = 0.002), FFM (42.01 kg ± 4.18 x 45.19 kg ± 4.74, p = 0.0059), FM (19.14 kg ± 5.26 x 21.44 kg ± 5.82, p = 0.0273), BMR (997.70 kcal/d ± 202.72 x 1318.20 kcal/d ± 157.58, p = 0.0117), Leptin (78.05 ± 76.25 x 236.86 ± 214.93, p = 0.0195), HOMA-IR (0.70 ± 0.41 x 1.92 ± 1.41, p = 0.0059) and WC (75.90 cm ± 7.52 x 79.49 cm ± 7.75, p = 0.002), with RQ significantly reduced (0.95 ± 0.12 x 0.77 ± 0.04, p = 0.002).

**Table 3:** Characteristics of DMPA group subdivided by weight gain after12 months

	Baseline	12 months	+ P value	Baseline	12 months	+ P value
	
	< 3 kg (n = 7)			> 3 kg (n = 10)		
	
	Mean ± SD	Mean ± SD		Mean ± SD	Mean ± SD	
Weight	61,24 ± 10,38	62,51^c^ ± 9,95	0,0156	61,1 ± 8,63	66,63^d^ ±8,48	0,002
BMI	23,44 ± 4,25	23,93^c^ ± 4,15	0,0313	23,47 ± 3,50	25,55^d^ ± 3,46	0,002
BS	1,64 ± 0,12	1,66^c^ ± 0,12	0,0313	1,64 ± 0,11	1,71^d^ ± 0,11	0,002
% Fat	30,70 ± 5,58	29,30 ± 6,99	0,5625	30,87 ± 4,58	31,76 ± 6,12	0,7891
FFM	42,51 ± 5,32	43,94 ± 6,50	0,1406	42,01 ± 4,18	45,19^d^ ± 4,74	0,0059
FM	19,44 ± 6,07	18,57 ± 6,20	0,7813	19,14 ± 5,26	21,44^d^ ± 5,82	0,0273
BMR	1224,43 ±2 66,82	1334,29 ± 125,04	0,1563	997,70 ± 202,72	1318,20^d^ ± 157,58	0,0117
RQ	0,87 ± 0,09	0,77^c^ ± 0,03	0,0313	0,95 ± 0,12	0,77^d^ ± 0,04	0,002
Adiponectin	10,20 ± 3,21	8,60 ± 4,82	0,375	5,89 ± 1,90	5,96 ± 2,80	0,8457
Leptin	133,53 ± 156,77	174,20 ± 184,10	0,2969	78,05 ± 76,25	236,86^d^ ± 214,93	0,0195
Visfatin	44,78 ± 10,82	45,76 ± 21,36	0,8125	50,68 ± 31,83	43,81 ± 16,23	0,4316
IL-6	1,30 ± 0,42	1,91 ± 1,80	1	1,12 ± 0,77	2,02 ± 2,71	1
TNF-α	4,70 ± 2,11	4,28 ± 3,33	0,2969	4,31 ± 1,98	3,62 ± 1,67	0,1934
HOMA-IR	1,25 ± 1,44	1,15 ± 0,84	0,5781	0,70 ± 0,41	1,92^d^ ± 1,41	0,0059
WC	74,14 ± 8,35	75,67 ± 8,97	0,125	75,90 ± 7,52	79,49^d^ ± 7,75	0,002

+: Wilcoxon test. Statistical significance: p < 0,05. c: Difference sub-group weight gain < 3 kg comparing baseline and after 12 months. d: Difference sub-group weight gain > 3 kg comparing baseline and after 12 months.

FFM: free fat mass; FM: fat mass; BMR: basal metabolic rate.

## DISCUSSION

We can consider that our population has a differential in relation to other studies evaluating the effect of DMPA, considering that our group was paired with the control group at baseline for BMI (± 1 kg/m^2^) and age (± 1 year), becoming a homogeneous group. Our study found that after 12 months of follow-up the injectable DMPA users had significant weight gain (3.01 kg). As most prospective studies evaluating the effect of DMPA were for at least 30 months ( 6 , 8 , 9 ), we can consider that the follow-up period of our study is short compared to the others, but we can already observe significant changes in important variables studied.

To better assess changes in weight and body composition, we made a subdivision of the DMPA group for weight gain (< 3 kg and > 3 kg), and observed significant changes in weight and body composition in both groups, but the group which gained > 3 kg had the more pronounced changes. This group had a mean gain of 5.53 kg, increased fat mass (2.3 kg) and fat-free mass (3.18 kg) after 12 months follow-up. The increase in fat-free mass in users of DMPA may be explained by the androgenic effect of this contraceptive method ( 13 ).

Berenson and Rahman ( 9 ), to compare changes in weight and body composition between different contraceptive methods, observed at 24 months of follow-up an increase of 4.4 kg and after 36 months a gain of 5.1 kg in the DMPA group. Of the 5.1 kg that the volunteers put on after 36 months, 4.1 kg were of fat mass. Moreover, a significant increase in total body lean mass after 30 months of follow-up was observed.

The main contributing factor to the increase in body weight after use of DMPA reported in most studies is the increase in fat deposits. After 30 months of follow-up of users of medroxyprogesterone, Clark and cols. ( 8 ) observed a gain of 6.1 kg, which is fully represented by increased fat mass. There are two other possible mechanisms that might explain the weight gain associated with DMPA use: the first is its glucocorticoid-like activity, which appears to be associated with increased fat, including visceral fat ( 14 ). However, secondly, it is also possible that the state of hypoestrogenism induced by the injectable may be another factor responsible for the increase in weight and body fat ( 15 ).

In our study, the subgroup that gained more weight at the end of the study also had a significant increase in waist circumference and HOMA-IR. Increased waist circumference is indicative of an increase in subcutaneous and visceral adipose tissue. HOMA-IR, although not yet characterized as insulin resistance (HOMA-IR > 2.7) in our group, indicates a probable trend for the development of insulin resistance with increased exposure time for the contraceptive method. Accumulation of excess fat can be a critical modulation factor for insulin sensitivity, since it promotes the release of free fatty acids into the adipocyte circulation ( 16 , 17 ). In addition, excess adiposity and adipocyte dysfunction result in dysregulation of adipokines, which may contribute to the development of various metabolic complications through altered glucose and lipid homeostasis as well as inflammatory responses ( 18 , 19 ).

In relation to changes in inflammatory markers, we observed a slight decrease in adiponectin levels, although it was not significant in the DMPA group. A greater reduction in the levels of this marker associated with weight gain and increased body fat can contribute to the development of insulin resistance. Moreover, there was a significant increase in leptin levels in the group with greater weight gain. Leptin is directly related to body fat in both experimental models and in humans ( 20 , 21 ), and is also related to food intake ( 22 ). These alterations relative to subclinical inflammation agree with those present in situations of obesity. The adipose tissue contributes to inflammation in both obese vascular and non-vascular tissues ( 23 ). Abnormal levels of fatty acids, lipids, cytokines and activate monocytes adipose tissue, increased secretion of inflammatory cytokines such as TNF-α, leptin, interleukin (IL)-1, IL-6, visfatin and reduced the secretion of anti-inflammatory cytokine adiponectin ( 24 ).

This study also has the important evaluation point of resting energy expenditure and respiratory quotient, given that data on basal metabolism with DMPA use are scarce, only two studies have assessed the resting energy expenditure in healthy women using DMPA ( 25 , 26 ). The basal metabolic rate increased significantly in the DMPA group and control. However, a more significant increase in this variable in subgroup DMPA, which gained more weight (> 3 kg), can be seen. This increase is mainly explained by an increase in fat mass and fat-free mass as well. In obese, the resting energy expenditure can be influenced mainly by fat-free mass, which is the largest contributing factor of variance of the basal metabolic rate (50-60%), and fat mass which represents (5-6%) of influence on the variation basal metabolic rate ( 27 ). The respiratory quotient of the two groups decreased after 12 months follow-up, indicating a change in metabolism of energy substrates. The volunteers at baseline had a characteristic respiratory quotient of high sugar metabolism but by the end of the study the profile changed with lipids starting to metabolize.

This study had the limitation of a small number of patients; including more patients could strengthen our results. Also we did not evaluate the lipid profile of the volunteers.

In conclusion, our study found significant changes in weight, body composition and metabolic profile of the population studied in the first 12 months of contraceptive use. These changes mainly increased body weight, leptin levels and HOMA-IR which can contribute to the development of some chronic complications, including obesity, insulin resistance and diabetes mellitus. According to the changes observed in our study, we suggest a more careful assessment of women who will start using the method as well as those who are already using this method of contraception. The exposure time method is considered the most important factor for the development of these changes.
